# Understanding developmental language disorder - the Helsinki longitudinal SLI study (HelSLI): a study protocol

**DOI:** 10.1186/s40359-018-0222-7

**Published:** 2018-05-21

**Authors:** Marja Laasonen, Sini Smolander, Pekka Lahti-Nuuttila, Miika Leminen, Hanna-Reetta Lajunen, Kati Heinonen, Anu-Katriina Pesonen, Todd M. Bailey, Emmanuel M. Pothos, Teija Kujala, Paavo H. T. Leppänen, Christopher W. Bartlett, Ahmed Geneid, Leena Lauronen, Elisabet Service, Sari Kunnari, Eva Arkkila

**Affiliations:** 1 Department of Otorhinolaryngology and Phoniatrics, Head and Neck Surgery, Helsinki University Hospital and University of Helsinki, Haartmaninkatu 4 E, 00029 HUS, POB 220 Helsinki, Finland; 2 Department of Psychology and Logopedics, University of Helsinki, Helsinki, Finland; 3 Department of Psychology and Speech-Language Pathology, University of Turku, Turku, Finland; 4 Research Unit of Logopedics, University of Oulu, Oulu, Finland; 5 School of Psychology, Cardiff University, Cardiff, UK; 6 Department of Psychology, City University of London, London, UK; 7 Centre for Advanced Research in Experimental and Applied Linguistics, Department of Linguistics and Languages, McMaster University, Hamilton, Canada; 8 Cognitive Brain Research Unit, Department of Psychology and Logopedics, University of Helsinki, Helsinki, Finland; 9 Department of Clinical Neurophysiology, Hospital for Children and Adolescents, University of Helsinki and Helsinki University Hospital, Helsinki, Finland; 10 HUS Medical Imaging Center, Clinical Neurophysiology, Helsinki University Hospital and University of Helsinki, Helsinki, Finland; 11 Department of Psychology, University of Jyväskylä, Jyväskylä, Finland; 12 Battelle Center for Mathematical Medicine, The Research Institute at Nationwide Children’s Hospital & The Ohio State University, Columbus, USA

**Keywords:** Language acquisition, Specific language impairment, Developmental language disorder, Sequential bilingualism, Event-related potentials, Clinical EEG, (Nonverbal) short-term memory, Artificial grammar learning, Child temperament, Child behavior, Genetics

## Abstract

**Background:**

Developmental language disorder (DLD, also called specific language impairment, SLI) is a common developmental disorder comprising the largest disability group in pre-school-aged children. Approximately 7% of the population is expected to have developmental language difficulties. However, the specific etiological factors leading to DLD are not yet known and even the typical linguistic features appear to vary by language. We present here a project that investigates DLD at multiple levels of analysis and aims to make the reliable prediction and early identification of the difficulties possible. Following the multiple deficit model of developmental disorders, we investigate the DLD phenomenon at the etiological, neural, cognitive, behavioral, and psychosocial levels, in a longitudinal study of preschool children.

**Methods:**

In January 2013, we launched the Helsinki Longitudinal SLI study (HelSLI) at the Helsinki University Hospital (http://tiny.cc/HelSLI). We will study 227 children aged 3–6 years with suspected DLD and their 160 typically developing peers. Five subprojects will determine how the child’s psychological characteristics and environment correlate with DLD and how the child’s well-being relates to DLD, the characteristics of DLD in monolingual versus bilingual children, nonlinguistic cognitive correlates of DLD, electrophysiological underpinnings of DLD, and the role of genetic risk factors. Methods include saliva samples, EEG, computerized cognitive tasks, neuropsychological and speech and language assessments, video-observations, and questionnaires.

**Discussion:**

The project aims to increase our understanding of the multiple interactive risk and protective factors that affect the developing heterogeneous cognitive and behavioral profile of DLD, including factors affecting literacy development. This accumulated knowledge will form a heuristic basis for the development of new interventions targeting linguistic and non-linguistic aspects of DLD.

**Electronic supplementary material:**

The online version of this article (10.1186/s40359-018-0222-7) contains supplementary material, which is available to authorized users.

## Background

### Background to the study

Language does not always develop as expected, which can have devastating effects on both individual and societal levels. Developmental language disorder (DLD, previously called specific language impairment, SLI) is a common developmental disorder comprising the largest disability group in pre-school-aged children. Approximately 7% of the population is expected to have DLD [1]. Somewhat surprisingly, DLD has received relatively little research interest compared to less prevalent disorders, such as autism spectrum disorders (ASD) and attention deficit/hyperactivity disorder (ADHD) [2]. Although DLD is diagnosed most often in childhood, the associated difficulties are not restricted to this developmental period. Rather, DLD also often leads to dyslexia [3] and it may continue to restrict the person’s social, academic, and occupational activities even beyond adolescence and into adulthood. For example, a recent study of adolescents in reform school found that poorer verbal skills were associated with elevated levels of later criminal behavior [4]. Further, the previous work of our research group has shown that 26% of adults with a childhood diagnosis of DLD in Finland are pensioned off and 19% live with their parents [5]. This truly highlights the long-term risk for social marginalization associated with DLD.

To cope with this risk caused by a developmental challenge, it is vital to understand better the interactions between harmful and protective factors that affect the developmental manifestation of DLD. However, at the moment, the specific etiological factors leading to DLD are not known. In many cases, developmental language difficulties are suggested to be caused by genetic factors [6]. At the neural level, perisylvian brain areas contributing to language processing are often affected [7]. However, the exact mechanisms that lead the neural abnormalities to cause DLD are not known. Presently, we do not even fully understand the range of cognitive or behavioral difficulties associated with DLD. For example, the cognitive difficulties have been suggested to span nonverbal as well as verbal domains, and the linguistic markers of DLD appear to vary from one language to another [8].

The genetic and neurobiological studies cited above suggest that DLD has a biological basis. However, language learning can be modulated also by, for example, reduced exposure to the language used in school and society. Of the population in Finland, 6.4% had a language other than Finnish, Swedish or Sami as their first language at the end of year 2016, and this percentage is rapidly growing [9]. Many of these are immigrants or people with immigrant background. Based on Finnish official statistics [10], one of the most significant predictors of successful employment for immigrants is an education acquired in Finland. Especially for bilingual children of immigrant families, language skills are the best predictors of successful educational attainment [11]. Naturally, also some of the bilingual children are expected to suffer from DLD. However, bilingual environment itself is not considered to be a risk factor for language impairment [12], and, thus, language impairment should be equally prevalent in monolinguals and bilinguals [13]. In contrast to this suggestion, of the children seen for the first time at the Audiophoniatric Ward for Children, Department of Phoniatrics, in the Helsinki University Hospital, a disproportionate 30–40% are multilingual. Although part of this amount may reflect a referral bias and challenges in diagnostics, it is also compatible with the possibility that the risk of language impairment, or especially severe language impairment [14], is elevated in bilingual and multilingual children compared to monolingual children. In annual follow-ups, the diagnoses of these bilingual children seldom change. This suggests that DLD does, indeed, explain their difficulties. This marked over-representation of bilinguals with suspected DLD warrants investigation of the underlying phenomena.

### Summary of the existing literature

#### Psychosocial factors in DLD

The child’s proximal environment, e.g. parent-child interaction patterns, and his or her individual traits and characteristics may affect both language development and response to intervention. For example, the quality of mother-child interaction moderates the effects of a biological disadvantage on later cognitive functioning [cf. studies on low birth weight, 15, 16]. In terms of temperamental traits, children with language difficulties have been shown to be less persistent in their temperament compared to typically developing (TD) peers [17]. However, to our knowledge there is no previous research on the effects of parent-child interaction specifically focusing on language development in DLD, nor has temperament been thoroughly assessed in a longitudinal setting.

Developmental language difficulties themselves may have a negative impact on the child’s self-esteem and well-being. Rescorla et al. [18] have shown that language delay is associated with social withdrawal already in toddlers, as assessed with the Child Behavior Checklist (CBCL) [19]. St Clair et al. [20] followed 7–16-year-old children with DLD with the Strengths and Difficulties Questionnaire (SDQ) [21] and found that during this time-period, social problems increased and emotional problems persisted into adolescence. In relation to the social problems, the previous work of our research team has shown that adults with a childhood history of DLD perceive many dimensions (usual activities, mental functioning, and speech) of their health-related quality of life (HRQoL) [22] to be poorer than that of the controls. This parallels with the fact that DLD adults of the study lived with their parents or were pensioned more often than the adult Finnish population on average [23]. We are not aware of any previous DLD research that has focused both on the etiological (e.g., temperament) and outcome psychological and psychosocial factors (e.g., well-being of the child). Recognizing these risk and protective factors and their consequences, both in the environment and within the child, would permit prevention.

#### Bilingualism and DLD

Differentiating DLD from TD in bilinguals is a challenging task for health care professionals. Lack of knowledge, normative data, and tools may often lead to over- or underdiagnosing. There are various suggestions for how DLD and bilingualism combine. Monolingual DLD and bilingual TD have been proposed to resemble each other in some ways, for example in terms of morphological forms used [24]. Bilingual DLD children have also been suggested to be affected by a double deficit [discussed in, 25], since they could suffer from both restricted cognitive (due to DLD) and restricted environmental (due to bilingualism) resources [see also, 26]. On the other hand, another recent suggestion is that although bilingual DLD children may suffer from restricted cognitive resources (similarly to monolinguals with DLD), the demands of their environment result in a “bilingual advantage” in, for example, executive functions [27]. Especially in the case of sequential bilingualism (L2 learning), it is suggested that various child-internal (e.g., first language, L1 typology, and child’s age) and child-external (e.g., amount of language exposure) factors play an important role in performance and development [27]. Despite of critically lacking information, such a large scale longitudinal study on 3–6-year-old bilingual DLD children has not been conducted.

#### Cognitive factors and DLD

Although DLD by definition means compromised skills in the language domain (domain-specific impairment), there is accumulating evidence that the difficulties of those with DLD may not actually be restricted to language, there instead being a domain-general impairment. In fact, nonlinguistic basic cognitive capacities are also likely to be involved, and some of these characteristics may well be shared across different languages. If this is so, new assessment and intervention possibilities could present themselves. Recent findings of domain-general capacities that might affect language development have been reported on different levels. At the etiological level, genetic factors behind DLD appear to affect not only language but also nonverbal ability [28]. At the cognitive level, there are several suggestions for nonlinguistic difficulties, for example, impaired general processing speed and short-term memory (STM) or working memory [29, 30]. One other recent hypothesis at the cognitive level, as put forward by Ullman [31], Nicolson and Fawcett [32], suggests that DLD could result from a generalized difficulty in acquisition of automatic skills, including procedural learning. Procedural learning is typically implicit and refers to learning of habits, skills, and procedures [33] as opposed to knowledge that can be explicitly articulated. Procedural learning mechanisms might be linked to language development in complex ways. For example, both procedural learning and language development would be compromised if their underlying cognitive core capacities are impaired. Also, they could form a cluster of functions linked to one another in a correlative or causative way. Unraveling these relations would have far-reaching consequences for how specific we perceive various developmental and learning impairments to be and how those with difficulties should be supported.

Initial diagnoses of DLD are often complemented with findings of impairments related to literacy when the child reaches school age. In fact, reading disability or dyslexia is so common among individuals with DLD that it has been suggested to be another symptom of the same syndrome. However, there is controversy as to the extent and nature of overlap between DLD and dyslexia [34]. We have shown recently that difficulties of written language in adults (i.e., dyslexia) correlate with modality-general impairments in processing speed [35] and STM [36], and argued that these may both relate to underlying difficulties in the processing of information that requires attentional control of temporal binding. Another recent project led by Prof. Laasonen (https://www.helsinki.fi/en/researchgroups/project-dyadd) showed that adults with developmental dyslexia also have difficulties in nonverbal procedural learning [37]. Importantly, poor performance in these affected nonlinguistic areas of cognition was shown to be related to poor linguistic skills. As developmental dyslexia could be one of the possible developmental end-results of childhood DLD, it is vital to expand this research to DLD children, in order to validate the findings of the older age-groups in young children [38].

#### Electrophysiology in DLD

Continuous electroencephalogram (EEG) recording has been a routine procedure in DLD diagnostics. One of the reasons is the necessity to exclude serious conditions, such as the Landau-Kleffner syndrome [39]. Otherwise, the rationale for clinical EEG in DLD diagnostics remains unresolved. Some studies have found elevated amounts of epileptiform activity in EEG of children with DLD [40–44]. Other researchers have suggested that especially those with syntactic-phonological or syntactic-lexical difficulties would have abnormalities in continuous EEG recording [45, 46]. To our knowledge, there is only one longitudinal study on clinical EEG in DLD [47]. It failed to find significant associations between original epileptiform EEG and later language development in a very small group of children. Thus, it remains unclear, whether children with DLD, in general, have abnormal EEG findings or whether the abnormalities are confined to a specific subgroup or if EEG has predictive value on DLD in a longitudinal setting. Finally, the mediating role of comorbid conditions has not been resolved. For example, developmental coordination disorder [48] and ADHD [49] have been associated with EEG abnormalities.

#### Genes and DLD

Developmental language difficulties are in many cases affected by genetic factors. Half of the children with DLD have relatives with language difficulties and the concordance rate for monozygotic twins is higher than that for dizygotic twins [50]. At least three different genetic loci (DLD1 at 16q, DLD2 at 19q, and DLD3 at 13q21) and two genes that are expressed in the brain (CMIP and ATP2C2 in chromosome 16) have been suggested to contribute to DLD [6]. The exact role of these genes is not known but, in their review, Li and Bartlett [6] suggest that they could contribute to phonological STM. Also other DLD candidate genes (e.g., CNTNAP2 and BDNF), have been suggested to contribute to STM as well as to difficulties in verbal comprehension and expression. Importantly, all four replicated genes involved in DLD aetiology, ATP2C2, BDNF, CMIP, and CNTNAP2, have common genetic variants that occur in persons of European ancestry. These genes have not been assessed in the Finnish population, which has some minor genetic differences from the rest of Europe due to the relatively small number of founding members of the Finnish population that migrated to present day Finland 4000 years ago. Further, more detailed information about different risk alleles’ contribution to specific cognitive and linguistic factors has not been conducted in a longitudinal setup, especially involving bilingual children.

### Aims

We present here an ongoing project, the Helsinki Longitudinal SLI study (HelSLI, http://tiny.cc/helsli) that investigates DLD in preschool children at the etiological, neural, cognitive, behavioral, and psychosocial levels of analysis with an aim to answer the many open questions and to increase our understanding of the multiple interactive risk and protective factors that affect the developing heterogeneous cognitive and behavioral profile of DLD. HelSLI study consists of five subprojects.

#### HelSLI-psychosocial

HelSLI-psychosocial investigates how the child’s psychological characteristics (i.e., temperament) and proximal environment (i.e., parent-child interaction) influence DLD and response to rehabilitation in a longitudinal setting. HelSLI-psychosocial investigates also how DLD relates to the psychosocial characteristics and well-being of the children. We hypothesize that both child temperament and parent-child interaction include risk and protective factors for language development, and that DLD itself is a risk factor for the long-term well-being of a child.

#### HelSLI-bilingual

The bilingual children of the current study are early sequential bilinguals who acquire Finnish as their second language not from the birth but early on in kindergarten. We use a two-way design (TD/DLD x mono/bilingual, that is, MonoTD, BiTD, MonoDLD, and BiDLD), longitudinal approach as well as consider age and exposure effects and their interaction. Thus, we are able to answer many of the open questions [25]. We hypothesize, based on the literature [12, 26, 51] and our preliminary data, that children with bilingual background will have poorer language performance compared to monolinguals when using tests developed for monolinguals but fewer comorbid characteristics. Possible bilingual advantage might be seen in compensating the hypothesized double deficit of restricted environmental resources and restricted cognitive resources. This advantage might prevent bilingual DLD children from falling behind their TD bilingual peers and could be observed in various cognitively demanding tasks included in the clinical neuropsychological battery and HelSLI-cognitive, and also in different linguistic areas at later stages of the longitudinal setting. We also hypothesize, since DLD and TD can resemble each other in bilingual setting, that it would be more appropriate to compare BiDLD children to BiTD children and not to MonoTD children when assessing developmental language disorder.

#### HelSLI-cognitive

In HelSLI-cognitive, we aim to test nonlinguistic factors that could potentially be used in prediction, diagnosis, and intervention of DLD across languages, in this case, auditory and visual STM and artificial grammar learning (AGL) [52]. We hypothesize, based on our own previous research and recent literature cited above, that DLD children will have more difficulties than TD children in the nonverbal tasks of STM and AGL across modalities, when required to maintain, chunk, manipulate, and learn patterns. In addition, we can explore whether any impairment in AGL can be identified to specific types of information, for example, high frequency bigrams vs. whole exemplars vs. long range associations.

#### HelSLI-EEG

To our knowledge, there is scarcely previous neurophysiological or functional imaging research on bilingual children with DLD [see, however 53]. Also, in case of studies on monolingual DLD children, most of the research has been conducted either with newborns, school-aged children, or adolescents whereas there is less research on preschool-aged children. The HelSLI-EEG sub-project thus focuses on identifying neurophysiological markers of DLD in monolingual and bilingual children with EEG and offers data on DLD children in the age range of 3–6 years – a time during which language skills develop rapidly but on which there is scarcely brain research. Both continuous clinical EEG and ERP recordings are being used. First, we aim to study, whether epileptiform activity is related to a specific cognitive impairment profile within DLD spectrum. Secondly, by ERP assessments, we aim to elucidate the cognitive dysfunctions in DLD at the levels of basic auditory processing, phonological processing, and STM as well as morphological processing. ERP assessments that are this wide-ranging have never been done in DLD research before. We preliminarily hypothesize that epileptiform abnormalities in clinical EEG are related to the severity of DLD in both mono and bilingual children. Based on previous literature on ERP indices in DLD, we expect to find attenuated MMN responses for tone frequency changes as well as consonant contrasts in syllable stimuli [54]. Importantly, we will be able to anchor these findings to other simultaneously measured linguistic and non-linguistic ERP contrasts, as well as to the detailed cognitive and linguistic behavioral profiles of individual children with DLD. In the framework of procedural learning impairment hypothesis, we expect to find indices that reflect neural dynamics of the acquisition of phoneme and morpheme sequences to be impaired in DLD.

#### HelSLI-genetic

HelSLI-genetic investigates the role of four known genetic risk factors (ATP2C2, BDNF, CMIP, and CNTNAP2) in DLD in the Finnish monolingual and bilingual populations. Should these genes be associated with DLD or related cognitive functions and neurophysiology in Finnish DLD cases, this will be the first such demonstration in this population, and these markers will be assessed for utility in predicting intervention outcomes. Also, these markers are of potential use as covariates for the analysis in the other subprojects, since the genetic markers may demarcate some error variance if multiple different DLD etiologies are, in fact, present. We hypothesize that language ability and more specifically STM (here also nonverbal) will be related to the genetic background in our sample.

## Methods and design

### Design and setting

HelSLI study is realized at the Audiophoniatric Ward for Children, Department of Phoniatrics, Helsinki University Hospital. Healthcare professionals on the department work in multidisciplinary teams focused on the assessment and diagnosis of the children with DLD or suspected DLD. These include medical doctors specializing in phoniatrics, speech and language pathologists, neuropsychologists, occupational therapists, special education teachers, and nurses. Most of the DLD sample data was gathered alongside normal clinical work. For the HelSLI study participants, we formulated standardized clinical EEG, neuropsychological (Additional file 1: Appendix 1) and speech and language assessment protocols (Additional file 2: Appendix 2) that were applied for each incoming and eligible first-time child at the Audiophoniatric Ward for children, Department of Phoniatrics, Helsinki University Hospital, during years 2013–2015.

Data collection begun in January 2013. The total number of 3-to-6-year-old children with suspected DLD who entered the HelSLI study was 246 (three entry years, 2013–2015) and those who fulfilled the inclusion criteria 227. The DLD children will be followed up during 2014–2018 on a yearly basis or less frequently, depending on whether they are monolinguals or bilinguals and what was their age when entering the study (see, Table 1). The last follow-up is before they enter school at the age of seven. The follow-up assessments are conducted mostly in the kindergartens. Children living outside the Helsinki metropolitan area are not followed-up unless they are assessed at Department of Phoniatrics for clinical purposes. Structured questionnaires are used for assessing the content and amount of intervention that takes place during the one-year periods between assessments. Separate questionnaires are sent to kindergartens and speech and language therapists.

**Table 1:** Structure of the HelSLI study: the schedule of enrolment, questionnaires and assessments

Timepoint			Pre-study		Onset											Follow-ups		
			t_00_		t_0_											t_1_	t_2_	t_3_
Group	Language	Age at Onset	Assessment															
			Informed consent	Background information	Genetic saliva sample	Neuropsychological	Speech and language therapist	STM and AGL tablet tasks	SDQ, ASEBA, CCC-2^a^	Medical examination	Clinical EEG	ERP	Video-taped play sessions	Temperament questionnaire	ALDeQ, ALEQ^b^	Speech and language therapist	Speech and language therapist	Speech and language therapist
DLD	Monolingual	3 yrs	✓	✓	✓	✓	✓	✓	✓	✓	✓	✓	✓	✓				✓
		4 yrs	✓	✓	✓	✓	✓	✓	✓	✓	✓	✓	✓	✓			✓	
		5 yrs	✓	✓	✓	✓	✓	✓	✓	✓	✓	✓	✓	✓		✓		
		6 yrs	✓	✓	✓	✓	✓	✓	✓	✓	✓	✓	✓	✓				
	Bilingual	3 yrs	✓	✓	✓	✓	✓	✓	✓	✓	✓				✓	✓	✓	✓
		4 yrs	✓	✓	✓	✓	✓	✓	✓	✓	✓				✓	✓	✓	
		5 yrs	✓	✓	✓	✓	✓	✓	✓	✓	✓				✓	✓		
		6 yrs	✓	✓	✓	✓	✓	✓	✓	✓	✓				✓			
TD	Monolingual	3 yrs	✓	✓	✓	✓	✓	✓	✓							✓	✓	✓
		4 yrs	✓	✓	✓	✓	✓	✓	✓							✓	✓	
		5 yrs	✓	✓	✓	✓	✓	✓	✓									
		6 yrs	✓	✓	✓	✓	✓	✓	✓									
	Bilingual	3 yrs	✓	✓	✓	✓	✓	✓	✓						✓	✓	✓	✓
		4 yrs	✓	✓	✓	✓	✓	✓	✓						✓	✓	✓	
		5 yrs	✓	✓	✓	✓	✓	✓	✓						✓	✓		
		6 yrs	✓	✓	✓	✓	✓	✓	✓						✓			

*DLD* developmental language disorder, *TD* Typically developing

Time points: t_00_ = enrolment; t_0_ = entering the study at age 3–6 years; t_1_, t_2_, t_3_ = yearly follow ups, last follow up at age 6. *STM* Short-term memory, *AGL* Artificial grammar learning, *SDQ* Strengths and difficulties questionnaire, *ASEBA* Achenbach System of Empirically Based Assessment, *CCC-2* Children’s communication checklist, *EEG* Electroencephalography, *ERP* Event-related potential, *ALDeQ* Alberta Language Development Questionnaire, *ALEQ* Alberta Language Environment Questionnaire

^a^Monolinguals: Parents and Kindergarten, Bilinguals: Kindergarten only

^b^Parent reports on first language development and language environment

In addition, 80 monolingual and 80 bilingual control children are recruited from the kindergartens of the metropolitan area of Helsinki, in order to gather normative information for the neuropsychological and speech and language tests for the sequentially bilingual children, as well as comparison data for the HelSLI subprojects. Control children are gathered from the same areas as DLD children and the proportion of girls versus boys per age group is compatible. The 3-and 4-year-old control children are followed up yearly, until they enter school, in order to define developmental pathways for both monolingual and bilingual TD children. In addition, bilingual 5-year-olds are also followed up until they enter school (see, Table 1). At the moment, all the DLD children have entered the study and are being followed up. Also, most of the TD children (over 150 of the total expected *n* = 160) have already been recruited to the study. Table 1 presents the general design of the HelSLI study. Below, the methods are described separately for each sub-project.

#### HelSLI-psychosocial

Temperament is parent-reported with the very short version of The Children’s Behavior Questionnaire (CBQ) [55]. Parent-child interaction is assessed with structured play sessions that are videotaped in order to evaluate both parenting and child behavior (1990 revision of the Erickson scales, [56]) and the dyadic level of the parent-child relationship [56, 57]. The ways that DLD relates to the psychosocial characteristics and well-being of the children are as assessed with questionnaires Child Behavior Checklist (CBCL) and the Teacher Rating Form (TRF), both part of the Achenbach System of Empirically Based Assessment (ASEBA) [19] and The Strengths and Difficulties Questionnaire (SDQ) [21].

#### HelSLI-bilingual

Speech and language development is investigated in Finnish, with the same standardized speech and language and neuropsychological test battery in all the groups, that is, monolinguals with typical language development (MonoTD), monolinguals with impaired language development (MonoDLD), and bilinguals with typical (BiTD) and impaired language development (BiDLD; see, Additional file 1: Appendix 1 and Additional file 2: Appendix 2). Because of the difficulties in assessing the first language of the bilingual children directly, with or without the help of an interpreter, we implement additionally indirect measures. In the HelSLI-bilingual, these are parent reports on the first language development (The Alberta Language Development Questionnaire, ALDeQ) [58] and the language environment questionnaire (The Alberta Language Environment Questionnaire, ALEQ) [59], which have been translated for the present research in collaboration with Professor Johanne Paradis, University of Alberta, Edmonton, Canada.

#### HelSLI-cognitive

STM capacities are assessed by asking children to make same/different judgments of small sets of non-linguistic stimuli (pictures or vocalizations of made-up animals), to measure the number of items each child can hold in memory. Nonlinguistic stimuli are used in order to assess memory functions independently from children’s language ability. These tests assess STM for visual and auditory stimuli distributed sequentially. Implicit learning abilities are assessed with AGL tasks [52] which first show children training examples of small sets of stimuli (similar in nature to those used for the STM tasks), and then ask children to classify novel sets of stimuli as being either “Good” or “Not good” with respect to the presumed pattern exemplified by the training items. These tools were built on the Graphogame literacy training platform (http://graphogame.com).

#### HelSLI-EEG

Continuous EEG is recorded during routine clinical checkups at the Department of clinical neurophysiology following clinical standards. Children are sleep deprived and EEG is recorded during a short daytime nap as well as during standard flashlight sequence procedures. During clinical routine EEG assessment, also a tone multifeature MMN paradigm, developed by Näätänen et al. [60] is used to measure the auditory discrimination profile, which has been shown to be a useful tool for investigating developmental disorders [54, 61–63]. The paradigm includes simultaneous measurements for tone frequency, duration, intensity, location, and gap contrasts. Some of the children with DLD and their controls, are invited to participate in more detailed ERP experiments in Cognitive Brain Research Unit, University of Helsinki [64]. One paradigm allows one to compare basic auditory processing efficiency of different sound features with speech specific sound processing, and thus gives novel insight on the specific neural dysfunctions associated with DLD at the individual level. The second ERP paradigm aims to track the neural circuitry and function needed in morphological processing [65]. Morphemes are the basic building blocks of the language meaning, and difficulties especially in word inflection have been proposed to be one of the core problems in DLD. This novel paradigm will now be used in children for the first time. Together all of these ERP paradigms allow specifying neurophysiological indices associated with cognitive dysfunction in DLD at the levels of basic auditory processing, phonological processing, and STM as well as morphological processing. This multilevel approach is particularly important as it allows the development of more reliable individual level indices and their comparison with cognitive and genetic measures of the HelSLI.

#### HelSLI-genetic

DNA in the HelSLI-genetic is extracted from saliva and analyzed by the international collaborators. Two sets of DNA markers are assayed. The first is a set of single nucleotide polymorphism (SNP) markers that constitute a DNA “barcode” that are unique across the population and are used for sample tracking and to assess relatedness among individuals [66]. That same set of SNPs was chosen to be ancestrally informative to provide information on continental genetic background to statistically control for admixture [66] across the control and DLD groups. A second set of SNP markers will provide information about common variation in the four (known) DLD genes. Analysis consists of methods previously deployed on similar datasets [67]. Briefly, ancestrally informative markers are analyzed by principal component analysis to provide a genomic summary of ancestry. We have shown that it is important to use the first three principal components as a covariate to reduce false positive associations across groups caused by random differences in ancestry [66]. The main genomic effects are modeled along with other variables in the regression framework using dummy coding to represent each of the three genotypic groups (AA, AB, BB; where A generically refers to the common SNP variant, and B generically refers to the more rare variant of the two).

### Characteristics of participants

The HelSLI study recruited four groups, that is, monolingual DLD (MonoDLD), bilingual DLD (BiDLD), monolingual TD (MonoTD), and bilingual TD children (BiTD). DLD children came from the Audiophoniatric Ward for children, Department of Phoniatrics. The TD children were gathered from kindergartens around the greater Helsinki area. In general, all four groups participate in all the subprojects of HelSLI, that is, psychosocial, bilingual, cognitive, EEG, and genetic (for exceptions, see Table 1).

Inclusion criterion for the DLD children was a referral to the Audiophoniatric Ward, Department of Phoniatrics, with a continuing concern in language development (in bilinguals in both languages) with no known biomedical etiology [68] (see Table 2 for sample description). Parent interviews and/or language assessment with the help of interpreter on first language (L1) had to confirm severe challenges in child’s first language. The children had a prior SLT assessment/intervention period in primary health care. They had normal hearing and no gross neurological findings, and had participated in routine follow-ups in local health-centers. In the ward, a medical examination, including ear-nose-and-throat (ENT) areas, gross and fine motor skills roughly, and a brief gross neurological status to rule out major findings or signs of any syndrome, was performed.

**Table 2:** Sample description

	Typical development	Language impairment
Monolingual • Finnish	N_MonoTD_ = 80	N_MonoDLD_ = 136
Bilingual • L1 not Finnish • L2 Finnish (≥ 1 yr exposure to Finnish in kindergarten)	N_BiTD_ = 80	N_BiDLD_ = 91
Recruited from	Kindergartens	Department of Phoniatrics
Exclusion criteria	• PIQ < 85 • Difficulties in language acquisition or other development ∘ Suspected or diagnosed in child ∘ Diagnosed in parents or siblings	• PIQ < 70 • Diagnosed neurological impairment or disability • Hearing impairment • ASD • Oral anomalies
Speech and language therapy	Short guidance on individual speech sounds allowed	SLT assessment or intervention required

In most cases, the DLD children are analyzed as one group, that is, we do not differentiate between, for example, receptive and receptive-expressive groups. However within the DLD children, a group with severe speech production problems on phonology/speech sound level is separated, since severe disorder in speech production may affect speech intelligibility and by implication expressive language (e.g. expressive vocabulary and sentence production). This distinction was necessary to make because in the Finnish ICD-10 [69] system speech sound disorders (such as CAS, childhood apraxia of speech) are included in SLI or DLD (ICD-10 diagnosis of F80.1). Classification for children with or without severe speech production problem based on difficulties at the phonological or speech sound level was made by combining the results from Finnish test of phonology (Fonologiatesti) [70] and speech and language therapist’s clinical report. In the Phonology test, the child had to perform below 12. percentile on phonotactic skills and in relation to age she/he had to have a significantly small phoneme inventory and/or severe difficulties in combining phonemes. If inclusion to the speech production problem group was made based on small phoneme inventory, omitted or substituted phonemes needed to be more than two and they had to be other than late emerging phonemes /r/ and /s/ or phonemes used only in loanwords. Children who did not produce speech at all were considered as their own group in some analyses.

Exclusion criteria for the DLD group were hearing impairment, intellectual disability, ASD, oral anomalies, or a diagnosed neurological impairment or disability (e.g., epilepsy, chromosomal abnormalities). The DLD children were required to have a performance Intelligence quotient (PIQ) of at least 70 [71]. For research purposes, the DLD group was divided into those who had PIQ in the range 70–84 and 85 or above. However, we did not require a mismatch between the verbal and nonverbal ability and we acknowledged the fact that DLD can co-occur with other neurodevelopmental disorders [68].

The TD children were gathered from kindergartens around the greater Helsinki area. They were required to not have difficulties in any of their languages or no intervention after an assessment. Guidance or short intervention period focusing on articulation, i.e. individual speech sounds, were not considered as exclusion criteria. The parents of TD children were required not to report any of the exclusion criteria and the TD children were required to have PIQ of at least 85 [71]. Further, exclusion criteria for the TD children were suspected or diagnosed difficulties in language acquisition or other development as well as diagnosed difficulties in these areas in parents or siblings.

Monolingual participants were required to have Finnish as their only home language. Sequential bilingual children vary in their first language (L1), but were required to have only one language at home (not Finnish, Swedish, or Sami). L1 languages in bilingual TD children were compatible to the ones of DLD children. Bilingual children had to have had at least one year of regular exposure to Finnish language in kindergarten. There are no standardized tests nor normative info on sequential bilingual performance in Finnish language-related tests. Therefore, we could not establish clear cut-off criteria for the test performance of the participating groups.

### Statistical analyses

A priori power analyses with G*Power [72] and RMASS (http://www.rmass.org/) were conducted to estimate appropriate sample sizes. For various research questions of subprojects guesstimates for the effect size varied along with the other aspects of power analysis. Detailed descriptions go beyond the scope of this paper, but two examples are given. For one age group (that is, e.g., 3 years old) an effect size as Cohen’s *d* = 0.6 was used for independent samples two-tailed t-test between DLD and TD children with α = .05 and 1 - β = .80 (power) using sample ratio N_DLD_ / N_TD_ = 0.67. This calculation resulted in N_DLD_ = 56 and N_TD_ = 38 for each age. The total number of participants recruited approximates these values (227 with suspected DLD, plus 160 TD across the four age groups). As another example, we computed the sample size for two-level mixed-effects linear regression model for the analysis of longitudinal data using the aforementioned values for α, 1 - β and sample ratio, four time points with AR1 error variance = 1.0 and *r* = .5, last time point mean difference = 0.6, 5% attrition rate, person variance components (intercept = 1.0, covariance = 0.1, slope = 0.1), and group × time interaction = 0.2. Here total number of subjects was 353. Again, the number of participants recruited (227 + 160 = 387) approximates the number indicated by the power analysis.

With large dataset and different subprojects, several different analytical lines will be pursued contingent upon the particular research questions of each subproject. Subsequent publications will describe details of the analysis used in each of them and only general tactics will be illustrated here. When all t_0_ (onset, baseline) assessments are finished, cross-sectional analyses will be carried out to explore relationships between variables of interest in each subproject. These analyses will include, e.g., different general linear modelling, multivariate analysis, and structural equation modelling techniques. In specific research questions, also generalized modelling may be used. As t_1_, t_2_, and t_3_ (follow-up) data is complete, longitudinal analysis (especially pertinent in HelSLI-bilingual) will be conducted. For this, multilevel modelling techniques for longitudinal data will be applied.

Both frequentist and Bayesian approaches to inference will be utilized depending on research questions of each subproject. In the former case, two-tailed nominal *p*-value of .05 and 95% confidence interval and, in the latter case, informative priors, when realizable, and 95% credible interval will be generally used.

## Discussion

**Fig. 1:**
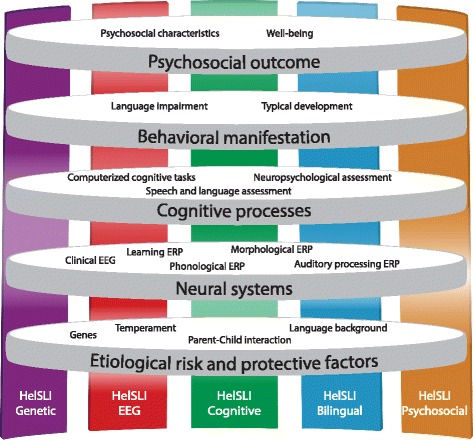
Levels of the study and description of HelSLI subprojects

Following the multiple deficit model of developmental disorders put forward by Pennington [73], the HelSLI subprojects investigate the DLD phenomenon at multiple levels of analysis: genetic and environmental etiological, neural, cognitive, behavioral, and psychosocial (see Fig. 1). The main aim of the project is to increase our understanding of the multiple interactive risk and protective factors that affect the developing heterogeneous cognitive and behavioral profile of DLD. Data collection is in active stage and the collected data will be unique in the world in its quality and quantity.

At the level of etiological risk and protective factors (see Fig. 1), we will be able to investigate the associations between biology (genes, temperament) and environment (parent-child interaction and language background) and use this knowledge, for example, to predict intervention outcomes and as covariates at other levels of analysis. At the level of neural systems, we will be able to investigate the neurophysiological correlates of DLD (both continuous EEG characteristics and ERP responses to various linguistic and non-linguistic auditory stimuli), evaluate the usefulness of EEG/ERP in individual diagnostics, and map these findings to the etiological level of analysis. We can determine, for example, the associations between genetic and language background and brain electrophysiology.

At the level of cognitive processes, we will be able to investigate the difficulties in nonlinguistic basic cognitive capacities that are expected to affect DLD across different languages with the aim to use this knowledge to develop language-independent tools for prediction, diagnosis, and intervention of DLD and later dyslexia. As described in the Background section, genetic factors behind DLD appear to affect not only language but also nonverbal performance. Especially (nonlinguistic) STM and procedural learning will be of interest here, since these have been associated also with the etiological and neural levels of analysis. At the level of behavioral manifestation, we will be able to investigate the variation ranging from typical to severely impaired language development. This level of analysis will enable testing for and validating subgroups suggested by the other levels of analysis (e.g., EEG abnormalities emerging in those with comorbid difficulties). Last, at the level of psychosocial outcome, we will be able to investigate associations between the other levels and a child’s psychosocial characteristics and well-being. With all these levels of analysis, the HelSLI study will be in a unique position to define correlative and probabilistic or derivational causal relations and map developmental pathways (or trajectories) in a large longitudinal sample. Moreover, there is little previous research into the relationship between bilingualism and DLD, and none that spans all these levels of analysis.

As the project will be carried out in a clinical setting, traditional and experimental assessment and intervention methods can be employed as part of the research project, in order to provide the DLD children comprehensive services. This and the longitudinal design make it possible to distinguish between associated and causal factors. The results could be used to help predict language development and its difficulties across language environments. Based on the results of the assessments, the current project will provide means for targeting some of the possibly causative factors, not just the resulting symptoms, with, for example, the adaptive computerized interventions of HelSLI cognitive that can be individually tailored based on the differences at the etiological, cognitive, and behavioral levels of analysis. This kind of early intervention in the promotion of health and equality and prevention of marginalization is pivotal, since funding targeted at supporting learning during the early years of education results in better outcomes than that provided during the later years [74].

## Additional files

Additional file 1 Appendix 1 Neuropsychological assessment battery. List of neuropsychological assessments used in the study. (DOCX 81 kb)

Additional file 2 Appendix 2 SLT assessment battery. List of speech and language assessments used in the study. (DOCX 25 kb)

## Abbreviations

ADHD: Attention deficit/hyperactivity disorder
AGL: Artificial grammar learning
ALDeQ: Alberta Language Development Questionnaire
ALEQ: Alberta Language Environment Questionnaire
ASD: Autism spectrum disorders
ASEBA: Achenbach System of Empirically Based Assessment
Bi: Bilingual
CAS: Childhood apraxia of speech
CBCL: Child behavior checklist
CBQ: Children’s behavior questionnaire
DLD: Developmental language disorder
EEG: Electroencephalography
ENT: ear-nose-and-throat
ERP: Event-related potential
HelSLI: Helsinki Longitudinal SLI study
HRQoL: Health-related quality of life
L1: First language
L2: Second language
MMN: Mismatch negativity
Mono: Monolingual
NPS: Neuropsychological
PIQ: Performance Intelligence quotient
SDQ: Strengths and difficulties questionnaire
SLI: Specific language impairment
SLT: Speech and language therapy
SNP: Single nucleotide polymorphism
STM: Short term memory
TD: Typically developing
TRF: Teacher rating form
