# Supplementary material for: Understanding developmental language disorder - the Helsinki longitudinal SLI study (HelSLI): a study protocol
Source: BMC Psychol. 2018 May 21;6:24. doi: 10.1186/s40359-018-0222-7 (PMC5963016; doi:10.1186/s40359-018-0222-7)
Supplement: Supplementary file 1 — Appendix 1 Neuropsychological assessment battery. List of neuropsychological assessments used in the study. (DOCX 81 kb) [file 40359_2018_222_MOESM1_ESM.docx]

Appendix 1 – Neuropsychological assessment battery

Wechsler Primary and preschool test of intelligence III, WPPSI-III [1]

- Information
- Vocabulary
- Word Reasoning
- Block Design
- Matrix Reasoning
- Picture Concepts
- Object Assembly
- Coding
- Symbol Search
- Receptive Vocabulary
- Picture Naming

Nepsy II [2]

- Auditory Attention
- Visual attention
- Comprehension of Instructions
- Phonological Processing
- Memory for Designs
- Narrative Memory
- Sentence Repetition
- Imitating Hand Positions
- Visuomotor Precision
- Theory of Mind, contextual
- Design Copying

Wechsler Intelligence Scale For Children IV, WISC-IV [3]

- Digit span

Lukiva [4]

- RAN for objects

Leiter-R [5]

- Forward memory
- Reverse memory

References

1. Wechsler D: **WPPSI-III - Wechsler Preschool And Primary Scale Of Intelligence - Third Edition**. Helsinki: Psykologien Kustannus Oy; 2009.

2. Korkman M, Kirk U, Kemp SL: **Nepsy II - lasten neuropsykologinen tutkimus**. Helsinki: Psykologien Kustannus Oy; 2008.

3. Wechsler D: **WISC-IV - Wechsler Intelligence Scale For Children - IV**. Helsinki: Psykologien Kustannus Oy; 2010.

4. Puolakanaho A, Poikkeus A-M, Ahonen T, Aro M: **LUKIVA - Lukivalmiuksien arviointimenetelmä 4-5-vuotiaille lapsille**. Jyväskylä: Niilo Mäki Instituutti; 2011.

5. Roid GH, Miller LJ (eds.): **Leiter International Performance Scale - Revised**. Wood Dale, IL: Stoelting, Co.; 1997.
