# Supplementary material for: Understanding developmental language disorder - the Helsinki longitudinal SLI study (HelSLI): a study protocol
Source: BMC Psychol. 2018 May 21;6:24. doi: 10.1186/s40359-018-0222-7 (PMC5963016; doi:10.1186/s40359-018-0222-7)
Supplement: Supplementary file 2 — Appendix 2 SLT assessment battery. List of speech and language assessments used in the study. (DOCX 25 kb) [file 40359_2018_222_MOESM2_ESM.docx]

**Appendix 2 – SLT assessment battery**

Reynell Developmental Language Scales III – RLDS III [1]

Boston Naming Test [2]

Expressive One-Word Picture Vocabulary Test 4 (EOWPVT-4) [3]

Receptive One-Word Picture Vocabulary Test 4 (ROWPVT-4) [4]

The Finnish Phonology Test [5]

Morphology tasks [6]

Finnish Nonword Repetition Test (FNRT) [7]

Multilingual Assessment Instrument for Narratives (MAIN) [8]

Sentence Comprehension Test [9]

The Boehm Test of Basic Concepts [10]

Test of Word Finding -2 [11]

Oral and verbal motor assessment

Alberta Language and Development Questionnaire (ALDeQ) [12]

Alberta Language Environment Questionnaire (ALEQ) [13]

Children’s Communication Checklist -2 (CCC-2) [14]

References

1. Kortesmaa M, Heimonen K, Merikoski H, Warma M-L, Varpela V: **Reynellin kielellisen kehityksen testi (Reynell Developmental Language Scales III)**. Helsinki: Psykologien Kustannus Oy; 2001.

2. Laine M, Koivuselkä-Sallinen P, Hänninen R, Niemi J: **Bostonin nimentätesti (Boston Naming Test)**: Psykologien kustannus Oy; 1997.

3. Martin N, Brownell R: **Expressive One-Word Picture Vocabulary Test 4 (Finnish version: Kunnari & Välimaa, in validation)** Novato: Academic Therapy Publications; 2010.

4. Martin N, Brownell R: **Receptive One-Word Picture Vocabulary Test 4 (Finnish version: Kunnari & Välimaa, in validation)**: Novato: Academic Therapy Publications; 2010.

5. Kunnari S, Savinainen-Makkonen T, Saaristo-Helin K: **Fonologiatesti (The Finnish Phonology Test)**. Jyväskylä: Niilo Mäki Instituutti; 2012.

6. Kunnari S, Savinainen-Makkonen T, Leonard LB, Mäkinen L, Tolonen A-K, Luotonen M, Leinonen E: **Children with specific language impairment in Finnish: The use of tense and agreement inflections**. *Journal of child language* 2011, **38**:999-1027.

7. Kunnari S, Tolonen A-K, Chiat S: **Finnish Nonword Repetition Test (FNRT)**: The test was developed within COST Action ISO804; 2011.

8. Gagarina N, Klop D, Kunnari S, Tantele K, Välimaa T, Balciuniene I, Bohnacker U, Walters J: **Part I. MAIN: Multilingual Assessment Instrument for Narratives**. In: *ZAS Papers in Linguistics 56.* edn. Berlin: ZAS; 2012.

9. Korpilahti P: **Lausetesti (Sentence Comprehension Test)**: Language & Communication Care Oy; 2012.

10. Heimo H: **Boehmin peruskäsitetesti (The Boehm Test of Basic Concepts)**. Helsinki: Psykologien kustannus Oy; 1993.

11. Tuovinen S, Ahonen T, Westerholm J: **Sananlöytämistesti (Test of Word Finding - 2)**. Jyväskylä: Niilo Mäki Instituutti ja Haukkarannan koulu; 2008.

12. Smolander S, Laasonen M, Kunnari S, Service E: **Finnish version of Alberta Language and Development Questionnaire (ALDeQ)**: Retrieved 4.10.2017 from <http://www.hus.fi/en/medical-care/medical-services/phoniatrics/HelSLI_Longitudinal_SLI_study/Results/Pages/default.aspx>.

13. Smolander S, Laasonen M, Kunnari S, Service E: **Finnish version of Alberta Language Environment Questionnaire (ALEQ)**: Retrieved 4.10.2017 from <http://www.hus.fi/en/medical-care/medical-services/phoniatrics/HelSLI_Longitudinal_SLI_study/Results/Pages/default.aspx>.

14. Bishop D: **CCC-2 –Lasten ja nuorten kommunikaatiotaitojen kysely (Children’s Communication Checklist -2 / CCC-2)** Helsinki: Hogrefe Psykologien kustannus Oy; 2015.
